# Supplementary material for: An open-source reproducible chess robot for human-robot interaction research
Source: Front Robot AI. 2025 Apr 16;12:1436674. doi: 10.3389/frobt.2025.1436674 (PMC12040702; doi:10.3389/frobt.2025.1436674)
Supplement: Supplementary file 1 [file DataSheet1.docx]

Supplementary Material

This is the supplementary material for the paper: Zhang, R., De Winter, J., Dodou, D., Seyffert, H., & Eisma, Y. B. (2025). An open-source reproducible chess robot for human-robot interaction research. Frontiers in Robotics and AI. <https://doi.org/10.3389/frobt.2025.1436674>

**Part A. Comparison with existing chess robots**

Table A1. *A comparison between the OpenChessRobot and other chess robots designed for human-robot interaction (HRI), evaluating aspects such as perception, manipulation, reproducibility, interactive features, and main purpose. The selected works explicitly mention the use of the robot in HRI studies or list “human-robot interaction” among their keywords.*

| **Robot** | **Perception** | | **Manipulation** | | **Reproducibility** | | **Interactive Features** | **Main Purpose** |
| --- | --- | --- | --- | --- | --- | --- | --- | --- |
|  | Method | Evaluation | Method | Evaluation | Material | Code/dataset |  |  |
| Pereira et al. (2008), iCat | Relying on E-board | Not applicable | Relying on E-board | Not applicable | iCat robot,  E-board | Not available | Facial expressions | To study “the influence of embodiment in the enjoyment of a game” (p. 1253) |
| Sajó et al. (2011), Turk-2 | Via an on-top RGB camera | Not reported | Via a robot arm | Not reported | A camera,  a customized robot arm, and a standard chess set | Not available | Facial expressions, speech of fixed terms | To present a chess robot that can “communicate with the opponent” (p. 493) |
| Matuszek et al. (2011), Gambit | Via a RGB camera | Piece Recognition: 93.22% | Via a robot arm | Success rate: 91.6% (in three games) | A camera,  a customized robot arm, and a normal chess set | Not available | Not available | To present a chess robot “that can play physical board  games against human opponents” (p. 4291) |
| LC et al. (2021), Power chess | Memorizing the game based on previous moves | Not applicable | Via a robot arm | Not reported | Two robot arms | Not available | Gesture expressions | To study the human perception of robot-to-robot gesture expressions in chess scenarios |
| Mac et al. (2023) | Via an on-top RGB camera | Not reported | Via a robot arm | Not reported | A camera, a Reactor X150 robot arm, and a standard chess set | Not available | Not available | To constructing  a physically capable Chess-Playing system to  compete against the player |
| OpenChessRobot | Via a RGB camera | Piece Recognition: 96.52%~100% | Via a robot arm | Success rate with a pawn:  Center: 100%  Halfway:  75%  Edge:  31%; more results in the paper | A camera,  a Franka Panda robot arm, a customized gripper, a normal chess set | Open-source code,3D-print models, and dataset available. | Gesture expressions and generative speech for game analysis | To represent an open-source reproducible chess robot that can play physical chess games with humans, interpret the game position verbally, and provide feedback with gestures |

**Part B. Dataset creation**

A new dataset was collected by placing all the pieces (63 pieces total, one rook missing) on the board, and then taking photos from the default camera pose. The corresponding labels in FEN format were then created. After performing row movements, column movements, and rotations, each piece appeared at least once at each square on the board, with rotations. The procedure for creating the dataset is detailed below. The dataset and fine-turned models can be found at: <https://doi.org/10.4121/1cb5bf64-468e-462a-a82e-c847d88a7a86>

**Starting position**

The pieces were manually placed in the middle of the square. The camera pose was such that pieces often occluded each other. Each piece was placed so that its adjacent neighbors included both white and black pieces.


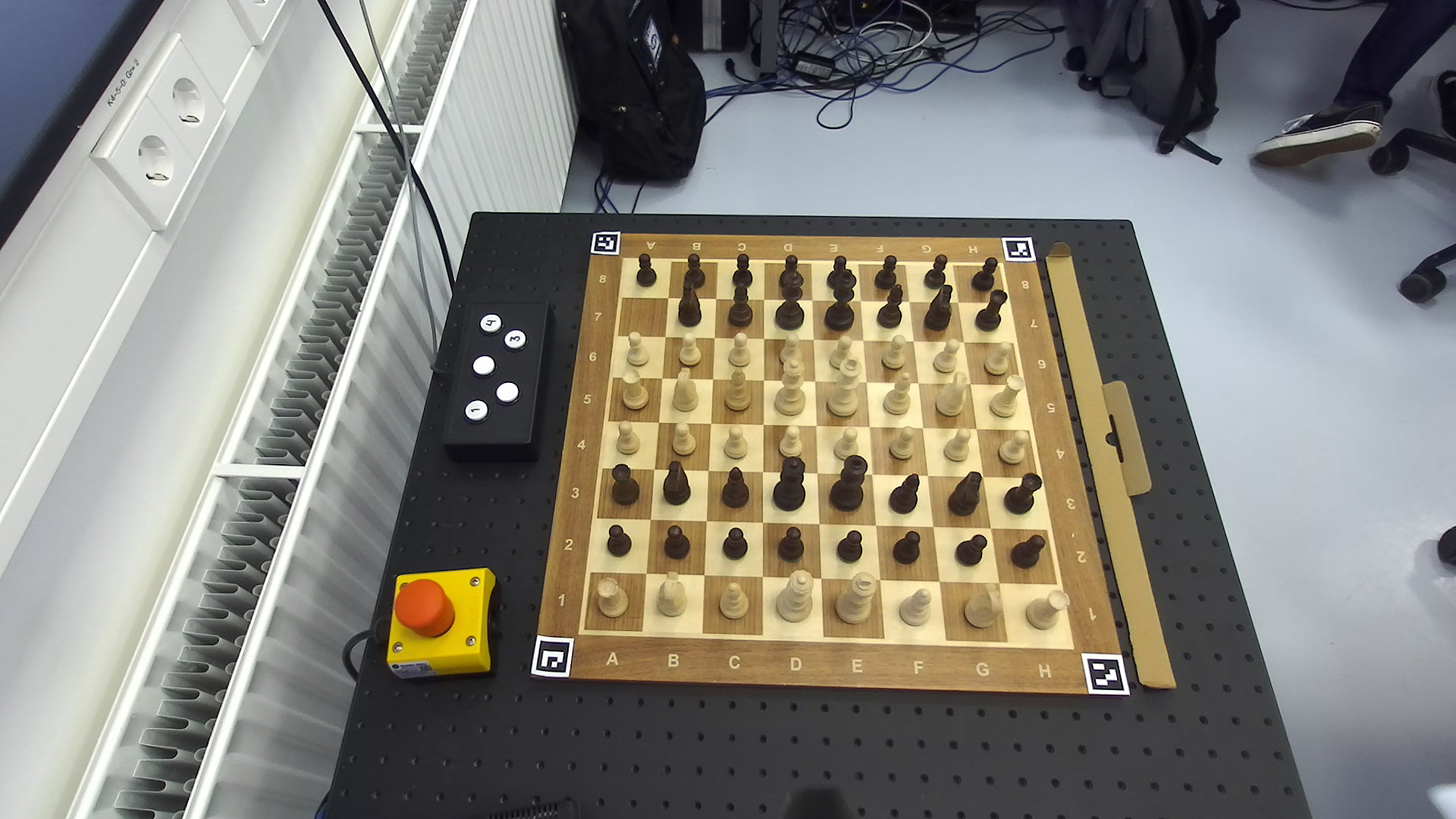


*Figure B1.* Starting position. Image in dataset: chess_image_data/raw/img/img_C0_R0_r0.png. Label in FEN: pppppppp/1nbqkbnr/PPPPPPPP/RNBQKBNR/PPPPPPPP/rnbqkbnr/pppppppp/RNBQKBNR

**Taking photos of the default and rotated piece positions**

At a game position, multiple photos were taken: one photo showed the default setting, and four more photos showed the pieces rotated. Four types of pieces were rotated because of their rotational asymmetry: white king, white knight, black king, and black knight. Kings were rotated twice, and knights were rotated four times to create different shapes of the pieces in the image. Each rotation was approximately 45 degrees.

| Default | Rotated |
| --- | --- |
| 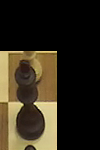 | 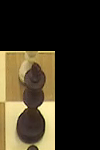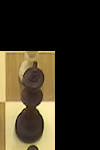 |
| 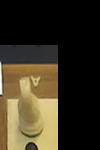 | 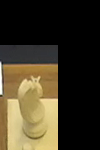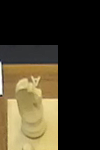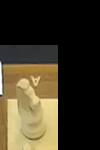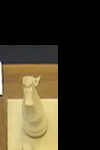 |

*Figure B2.* Illustration of piece rotation.

Following the above procedure, at each chess piece’s position, five images were taken (unrotated, both the knight and king rotated twice, knight rotated twice more). Photos are named as: img_CX_RY_[r0, r1, r2, r3, r4].png

**Translating the pieces by row**

In the direction of increasing rank, the rows of pieces were moved to make a new chess piece position. Then five photos were taken at the new position with the same method mentioned above. The row translation was repeated three times (R0 > R1 > R2 > R3).


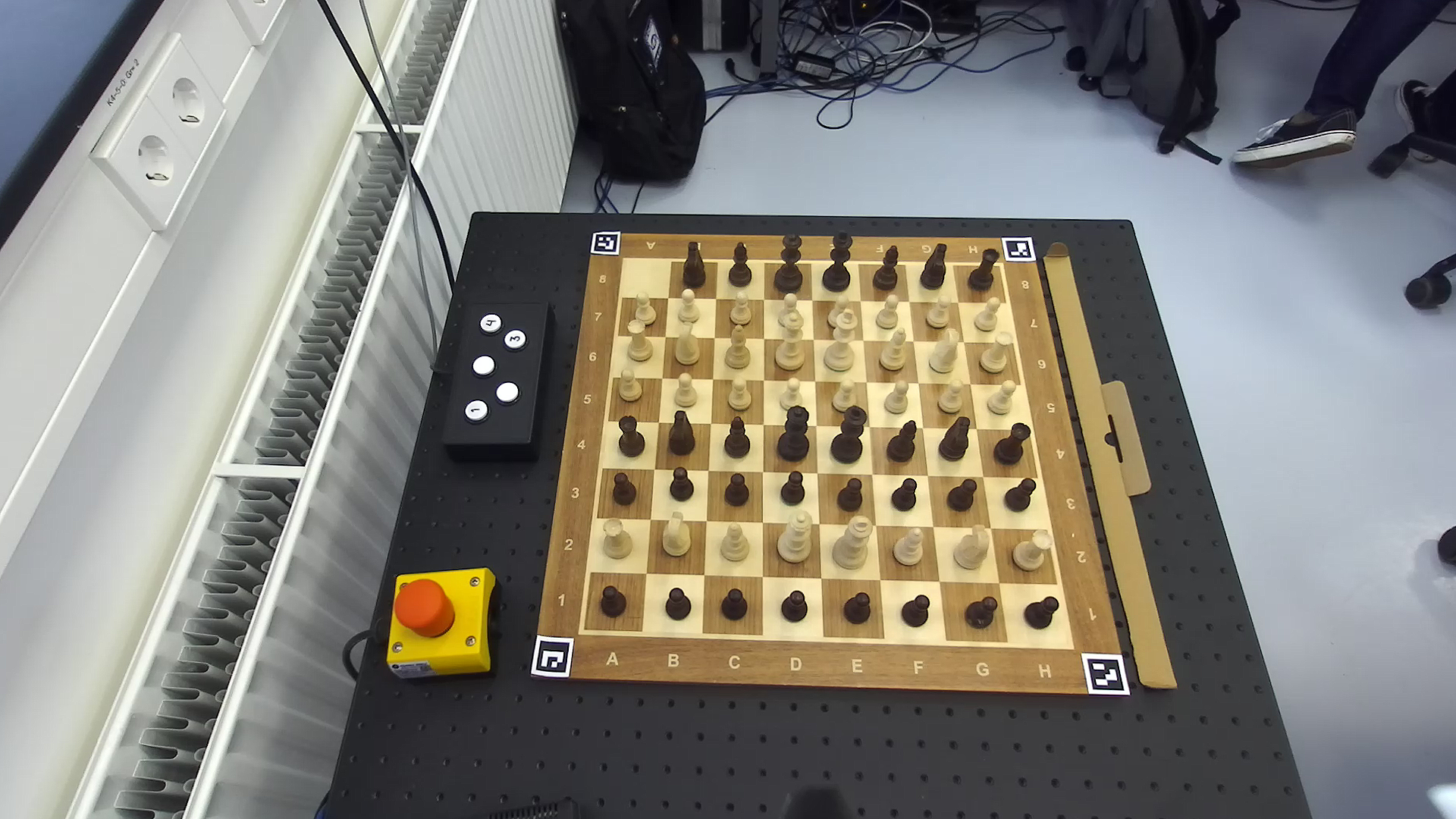


*Figure B3.* Starting position, after one upward row shift. Image in dataset: chess_image_data/raw/img/img_C0_R1_r0.png. Label in FEN: 1nbqkbnr/PPPPPPPP/RNBQKBNR/PPPPPPPP/rnbqkbnr/pppppppp/RNBQKBNR/pppppppp

**Translating the pieces by column**

After translating the row three times, one additional row translation was realized (R3 > R0), in order to restore the R0 game position. After that, a column translation in the (A > H) direction was performed (C0 > C1), followed by taking photos at this new game position using the same method mentioned above.


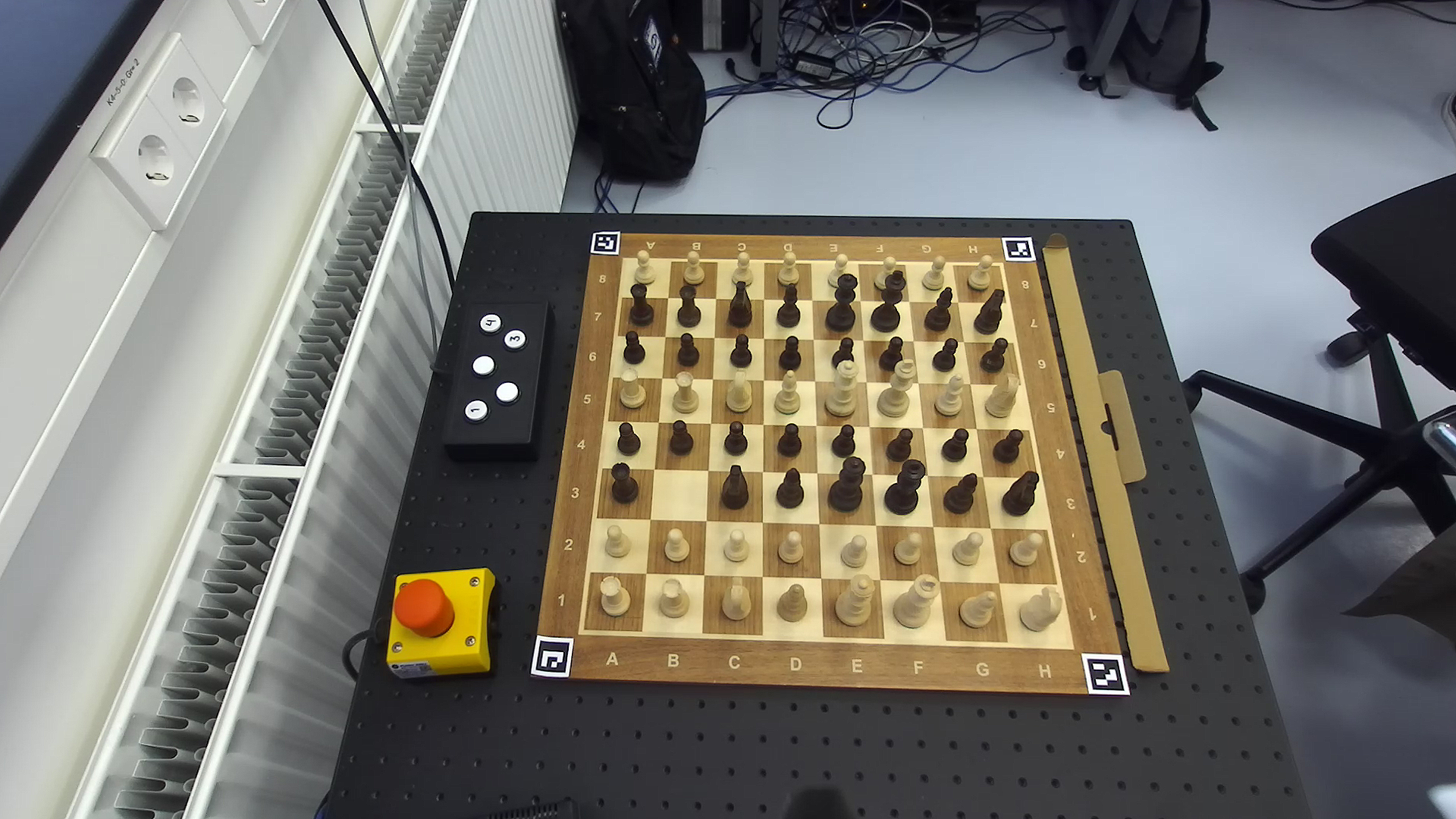


*Figure B4.* Starting position, after one rightward row translation. Image in dataset: chess_image_data/raw/img/img_C1_R0_r0.png. Label in FEN: PPPPPPPP/rrnbqkbn/pppppppp/RRNBQKBN/pppppppp/r1nbqkbn/PPPPPPPP/RRNBQKBN

**Creating the datasets**

A total of 160 raw images were collected (4 row movements × 8 column movements × 5 orientations), from which three datasets were created: default, rotated, and visually shifted.

The default dataset (**D)** contained 2048 sub-images of unrotated pieces extracted from the raw images, with every unique piece-square combination represented at least once.

The rotated dataset (**R)** contained 1280 sub-images of each rotated piece (black king, white king, black knight, and white knight) extracted from the raw images, with every unique piece-square-rotation combination represented at least once.

The visually shifted dataset (**S)** contained 8192 sub-images with cropping windows shifted in four directions (up, down, left, right). The displacement in each direction was ¼ of a square’s length. For each shift direction, 2048 sub-images were extracted from the raw images without piece rotations, following the same procedure used to generate the dataset **D** but with displaced cropping windows. The visually shifted dataset is used only for testing, not for training.

**Training data selection and train-val-test split**

Several squares were selected as key squares for the board. Sub-images of pieces on these squares were used to train the model; this model was then tested on the remaining squares to show its generalization ability. Two sets of key squares were tested:

1. 3×3:
   key_file: A, E, H;
   key_rank: 1, 4, 8;
   training set: 9 × 12 sub-images;
2. 4×4:
   key_file: A, E, D, H;
   key_rank: 1, 4, 5, 8;
   training set: 16 × 12 sub-images;

The training dataset (d) from the default dataset was generated using all sub-images from these key squares. One sub-image was chosen randomly for each square-piece pair.

The training dataset (r) was generated from the rotation dataset using all sub-images rotated by 90 degrees. One sub-image was chosen randomly for each square-piece pair with the same rotation angle.

Another training dataset (rr) was generated from the rotation dataset using all sub-images rotated by both 45 degrees and 90 degrees. For black and white knights, one sub-image was chosen randomly for each square per rotation angle.

Then, sub-images of the remaining squares were split into a validation set and a test set using a ratio of 20:80. The test sets corresponding to datasets d, r, rr, respectively, are referred to as d, r, rr.

Before feeding the training set to train the model, some random image augmentation was applied to each sub-image. These parameters were set through trial and error.

Table B1. *Parameters for augmentation.*

| **Attributes** | **Range** |
| --- | --- |
| brightness/hue/saturation | [-20%, 20%] |
| contrast | [-30%, 30%] |
| scale | [0.8, 1.2] |
| translation | [-0.03, 0.1] |
| Examples: | |
| 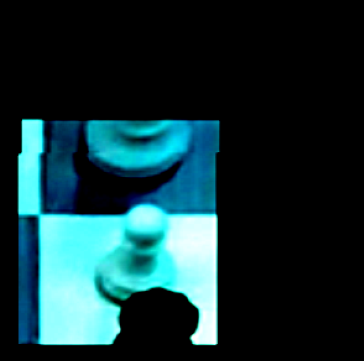 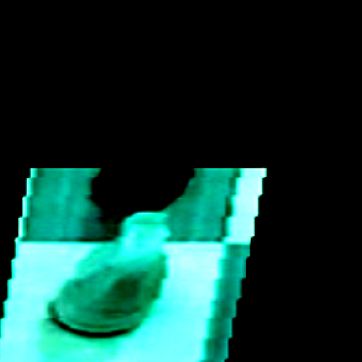 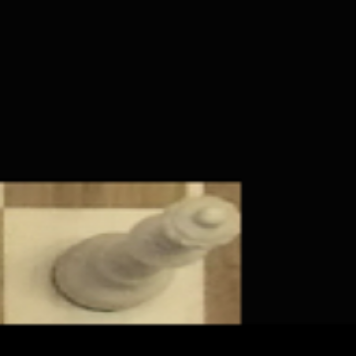 | |

**Part C. Links to videos of the OpenChessRobot**

Video 1: Demonstrations of the robot’s movements and game explanation. Human-robot gameplay with body posture feedback is also included. Link: <https://www.youtube.com/watch?v=RenXuiwX4Go>

Video 2: A sample from an annotated video recording using the Tobii Glasses 3 eye-tracker from the perspective of a professional chess player. To protect the privacy of the participant, audio has been removed. Link: <https://www.youtube.com/watch?v=j-b32ILQjtw>

Video 3: The three videos used in the online questionnaire survey. Link: <https://www.youtube.com/shorts/Mj_rZVboH9s>

Transcripts of video 3:

The *Robotics Education* scenario: *I determine the exact 3D position of the board first by detecting the four ArUco markers placed on it and calculate the chessboard’s 3D grid. Then once I identify which square each piece is on through my occupancy and piece classification, I map that square to its corresponding 3D coordinates on the board.*

The *Chess Coach* scenario: *Great, I am playing e4 which is one of the most popular and aggressive openings for White,controlling the center and opening lines for my queen and bishop. Now if Black plays c5 they are introducing the Sicilian Defense. Instead of directly placing a pawn in the center (like with e5), Black plays c5 to exert influence on the d4 square.*

The *Home Entertainment* scenario: *This is the famous position from 2001: A Space Odyssey, where Frank Poole faced against the HAL 9000. In the movie Poole as White captured on a6 with the queen and walked right into a forced mate. But if we analyze carefully White might try something safer—like shifting the queen to b7 instead of immediately grabbing the pawn. Or pushing a rook to e1. What would you like me to try?*

**Part D. A case study with an expert chess player**

On May 25, 2023, during development, we invited a FIDE Master to play against the initial version of the OpenChessRobot. His feedback was used to improve the interactive features, leading to the current version of the OpenChessRobot.

Two games were played between the human and the robot. In the first game, the player played against a Stockfish chess engine. In the second game, the player experienced the verbal feedback on the game positions, while the robot used the chess engine Maia ELO 1900 (McIlroy-Young et al., 2020), which was trained on human games. The player wore Tobii Pro Glasses 3 during the games to record the gaze activity and was instructed to think aloud (a video can be found in Supplementary Material Part C). An experimenter prompted the player to explain strategies and thoughts. After the games, an interview was conducted to gather feedback on the player’s experience with the robot, potential use cases, and suggestions for improvements.

The recordings were processed using AssemblyAI’s Speech-to-Text API for speech recognition and speaker diarization. OpenAI’s ChatGPT o1 model was used to translate the transcriptions from Dutch to English and to answer specific questions (see Table D1). Eventually, only the recording of the interview was analyzed, as most comments during the games were about the game strategy.

Table D1. *Questions to ChatGPT o1 and corresponding responses.*

| **Question 1**: What is the playing experience of the chess player playing against the chess-playing robot? Provide concise answers in bullet points.  **Answer from ChatGPT** **o1**:   - The chess player did not find the robot intimidating and felt it resembled playing a regular human opponent primarily due to its strong moves. - The novelty of a physical, tangible opponent added a different dynamic compared to playing chess online. - The player expressed that faster, more natural robot movements would make the experience more enjoyable. - The absence of meaningful feedback (e.g., identifying mistakes or strong moves) reduced the robot’s value as a training tool. - The player would find the robot more beneficial if it played and provided insights in a more human-like and instructive manner. |
| --- |
| **Question 2**: What does the chess player think about the possible use cases and the unlikely use cases of this chess robot? Provide concise answers in bullet points.  **Answer from ChatGPT** **o1**:   - Likely Use Cases:   - Practicing specific chess openings or positions.   - Receiving instructive feedback on moves and tactics.   - Serving as a training tool for beginners.   - Providing an entertaining, physical alternative to playing online. - Unlikely Use Cases:   - Playing random casual games where no human-like or instructive element is added.   - Replacing standard online engines for straightforward chess play.   - Serious professional training relying solely on a “perfect” engine (like Stockfish). |
| **Question 3**: What are the suggestions from the chess player to improve the robot? Provide concise answers in bullet points.  **Answer from ChatGPT** **o1**:   - Offer immediate feedback on moves, including identifying blunders or strong plays. - Employ a more human-like engine (e.g., the Maia engine) to create a realistic playing style. - Increase the speed and fluidity of the robot’s physical movements. - Provide instructive commentary that explains why certain moves are good or bad. - Allow scenario-based training, such as practicing specific openings or positions. |

From the interview, we learned that the player did not find the robot intimidating and recognized its potential use for beginners in practice or entertainment scenarios. However, he noted that the robot could be improved to play more like a human, both in terms of game strategy and motion speed. To improve the robot, he suggested it should provide direct feedback on move quality and move faster.

The eye-tracking data from the two games was processed into annotated videos, showing where the player was looking during the game. The gaze data provides insights into how the player anticipated the robot’s next move and how often the player engaged in gaze interaction with the robot’s physical body (see Figure D1). These gaze recordings offer valuable data for future HRI studies on this platform. A video clip of the eye-tracking data is included in Supplementary Material Part C (Video 2).


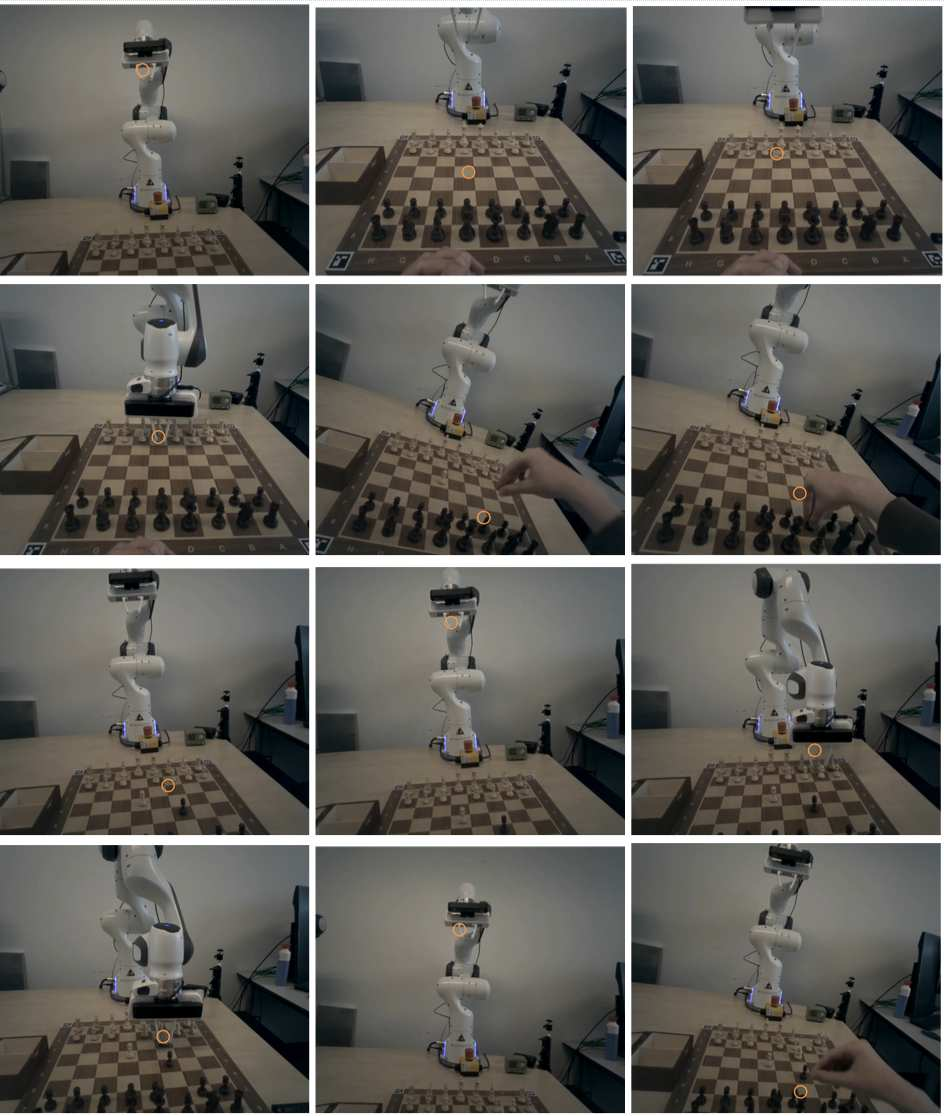


*Figure D1.* Annotated video with gaze overlay. Row 1 (left to right): The player looked at the robot and scanned the board for the robot’s possible moves. Row 2 (left to right): The robot made its first move, followed by the player’s first move. Row 3 (left to right): The player predicted the robot’s next move and looked at the robot, waiting for its execution. Row 4 (left to right): The robot executed its move, the player gazed at the robot again, then made the second move.

**Reference**

McIlroy-Young, R., Sen, S., Kleinberg, J., & Anderson, A. (2020). Aligning superhuman AI with human behavior. *Proceedings of the 26th ACM SIGKDD International Conference on Knowledge Discovery & Data Mining*, 1677–1687. <https://doi.org/10.1145/3394486.3403219>

**Part E. The full questionnaire**

| Category | Question |
| --- | --- |
| GPT familiarity | Q1. “*Have you ever heard of ChatGPT?”* (Yes, No, I prefer not to respond)  Q2. “*How often do you use ChatGPT?”* (Every day, 4-6 days a week, 1-3 days a week, About once every 2 weeks, About once a month, Less than once a month, Never, I prefer not to respond)  Q3. *“Do you use a paid version of ChatGPT (Plus or Pro)?”* (Yes, No, I prefer not to respond) |
| Video Demo | |
| Perception of the robot | Q4. (A/B/C) “*Do you think this robot could be used <in schools to explain to students how a robot works?> / <in schools to teach students chess?> / <at home to provide entertainment?>”* (Definitely not, Probably not, Neutral, Probably Yes, Definitely yes, I prefer not to respond)  Q5. “*Comment on the technical quality of the robot as shown in the video. Mention positive or negative aspects.”* (A response of at least 20 characters was required)  Q6. “*Do you have any recommendations about how to improve the robot?”* (A response of at least 20 characters was required)  Q7. (A/B/C) “*Do you think that within 10 years, robots like this could serve as a replacement of existing screen-based <computers to help understand technical topics such as computer vision?> / <computers to improve chess skills?> / <forms of entertainment such as television?> Please elaborate on why or why not.*” (A response of at least 20 characters was required)  Q8. “*Would you welcome a future where robots that can talk, equipped with artificial intelligence (such as improved versions of ChatGPT), replace human workers?”* (Definitely not, Probably not, Neutral, Probably Yes, Definitely yes, I prefer not to respond) |
| Demographics | Q9. Age, Q10. Gender, Q11. Education |
| Familiarity with the context of scenarios | Q12. *“Are you a technology enthusiast?”* (Not at all, Slightly, Moderately, Very, Extremely, I prefer not to respond)  Q13. *“Are you familiar with the movie 2001: A Space Odyssey?”* (Not at all, Slightly, Moderately, Very, Extremely, I prefer not to respond)  Q14. “*Did you ever play in an official FIDE-rated chess tournament?*” (No, Yes, I prefer not to respond)  Q15. *“Are you a chess enthusiast?”* (Not at all, Slightly, Moderately, Very, Extremely, I prefer not to respond) |

**Part F. Prompts for ChatGPT o1 and the raw outputs**

| Prompt to ChatGPT o1 to analyze Q5 | Based on the given answers to the question: “Comment on the technical quality of the robot as shown in the video. Mention positive or negative aspects.”, summarize the most mentioned three positive and negative aspects in the answers. Make concise bullet points. Answers: |
| --- | --- |
| Output of Scenario A | Most Mentioned Positive Aspects   - Smooth and Precise Movement: Many noted the arm moves fluidly, handling chess pieces accurately. - Clear Voice/Explanation: Several answers highlighted that the robot’s speech is generally understandable and coherent. - Advanced Technical Ability: Respondents frequently praised its strong board-detection and analytical capabilities.   Most Mentioned Negative Aspects   - Overly Technical/Complex Language: A common concern was that its explanations could be too advanced or confusing for some audiences. - Slow or Lengthy Process: Multiple people felt the scanning or movement took too long. - Robotic or Distracting Voice/Noise: The monotone sound and mechanical noise were cited as off-putting or not engaging. |
| Output of Scenario B | Most Mentioned Positive Aspects   - Smooth and precise movement: The robot accurately picks up and places pieces without disturbing others, and its motion control is generally fluid and stable. - Clear explanations: Many appreciated that it articulates moves clearly, providing reasoning and context for each chess move. - Effective teaching approach: Explaining why certain moves are played and discussing strategy was seen as a strong educational feature.   Most Mentioned Negative Aspects   - Robotic or unnatural voice: Several found the voice to be lacking a human touch or warmth, making it less engaging. - Noisy or distracting movements: The mechanical sounds during operation were frequently cited as disruptive. - Limited interactivity: Users noted a lack of two-way communication or the ability to ask questions, reducing the personalized learning experience. |
| Output of Scenario C | Most Mentioned Positive Aspects   - Smooth and precise movement (many note its fluid, accurate handling of chess pieces) - Intelligent or knowledgeable (demonstrates strong chess skills and offers insightful commentary) - Entertaining or interactive (engages the user through narration, voice, and conversation)   Most mentioned negative aspects   - Loud or noisy (the whirring and mechanical sounds are off-putting to some) - Bulky or large (the size and industrial look make it less home-friendly) - Slow or boring (some feel its pace and monotone delivery can be dull over time) |
| Prompt to ChatGPT o1 to analyze Q6 | Based on the given answers to the question: “Do you have any recommendations about how to improve the robot?”, summarize the most mentioned five recommendations in the answers. Make concise bullet points. Answers: |
| Output of Scenario A | - More natural, varied, or human-like voice   (e.g., less robotic, different voice options, more expressive tone)   - Simpler, age-appropriate explanations   (e.g., less technical jargon, clearer language for children)   - Friendlier or more appealing design   (e.g., adding a face or screen, making it look less mechanical and more welcoming)   - Reduced mechanical noise   (e.g., quieter motors, less distracting sounds during movement)   - Faster or smoother operation   (e.g., increased speed, more fluid arm movements) |
| Output of Scenario B | - More natural, simpler speech: Many respondents suggested using a less robotic voice and avoiding overly technical language, especially for beginners or children. - Reduce mechanical noise: A common recommendation was to make the robot’s arm and movements quieter so it’s less distracting. - Friendlier design and smaller size: Several mentioned the robot’s industrial look, proposing a more approachable or compact form—e.g., adding a face, giving it a name, or shrinking its footprint. - Greater interactivity and Q&A: Multiple answers emphasized an option for students to ask questions and receive explanations in real time. - Clearer movement cues: Suggestions included using a laser pointer or other visual indicators to highlight the squares being discussed. |
| Output of Scenario C | - Reduce mechanical noise: Many participants found the whirring and buzzing of the robot’s arm distracting and suggested quieter motors or motion. - Use a more natural, expressive voice: A large number of people felt the robot’s monotone voice should be improved, either by adding emotion, varying intonation, or offering different accents. - Make the design more compact: Several responses pointed out that the robot is bulky or tall, recommending a smaller, sleeker form factor. - Improve overall aesthetics/approachability: Suggestions ranged from adding a “face” or a friendlier exterior to changing color schemes and reducing exposed wires. - Enhance movement fluidity: Smoother, faster, or less “jerky” movement was mentioned to make the robot feel more natural and engaging. |
| Prompt to ChatGPT o1 to analyze Q7 – Scenario A | Based on the given answers to the question: “Do you think that within 10 years, robots like this could serve as a replacement of existing screen-based computers to help understand technical topics such as computer vision? Please elaborate on why or why not.”, summarize the most mentioned three concerns in the answers. Make concise bullet points (keywords and explanation in one sentence). Answers: |
| Output of Scenario A | - High cost and limited cost-effectiveness: Many responses cite that these robots would be too expensive or impractical to deploy at scale compared to existing screen-based tools. - Concerns about acceptance and human interaction: Several people doubt whether users would find a robot’s impersonal or purely mechanical explanations as engaging as human teachers or more familiar screen-based interfaces. - Incomplete or restricted functionality: Respondents worry that a robot arm cannot cover the breadth of content or flexibility offered by computers, and may not fully replace the versatility (e.g., code viewing, detailed visuals) screens currently provide. |
| Prompt to ChatGPT o1 to analyze Q7 – Scenario B | Based on the given answers to the question: “Do you think that within 10 years, robots like this could serve as a replacement of existing screen-based computers to improve chess skills? Please elaborate on why or why not.”, summarize the most mentioned three concerns in the answers. Make concise bullet points (keywords and explanation in one sentence). Answers: |
| Output of Scenario B | - High cost: Many respondents believe the robots would be too expensive for widespread adoption. - Limited portability: Several people noted that these physical robots are bulkier and less convenient than screen-based computers. - Reduced versatility: Others highlighted that a single-purpose chess robot cannot match the broader functionality and advanced analysis tools offered by standard computers. |
| Prompt to ChatGPT o1 to analyze Q7 – Scenario C | Based on the given answers to the question: “Do you think that within 10 years, robots like this could serve as a replacement of existing screen-based forms of entertainment such as television? Please elaborate on why or why not.”, summarize the most mentioned three concerns in the answers. Make concise bullet points (keywords and explanation in one sentence). Answers: |
| Output of Scenario C | - Lack of variety and visual/storytelling depth – Many respondents feel a robot cannot replicate the diverse content and creative appeal (shows, movies, storylines) that TV provides. - Preference for passive entertainment – A common concern is that people want to “sit and watch,” whereas a robot requires more active engagement and isn’t as effortless as simply turning on a screen. - Insufficient human/emotional element – Several mention that robots lack the emotional connection or relatable human factor that makes television and film compelling. |
| Prompt to ChatGPT o1 and Gemini 2.0 Flash Think to classify answers of Q7 – Scenario A | Based on the given answers to the question: “Do you think that within 10 years, robots like this could serve as a replacement of existing screen-based computers to help understand technical topics such as computer vision? Please elaborate on why or why not”, classify each answer to one of the three classes: [positive, neutral, negative] and add the category to each row. Add quotation marks if any answer is not in quotation marks. The answers are provided as a csv. |
| Prompt to ChatGPT o1 and Gemini 2.0 Flash Think to classify answers of Q7 – Scenario B | Based on the given answers to the question: “Do you think that within 10 years, robots like this could serve as a replacement of existing screen-based computers to improve chess skills? Please elaborate on why or why not”, classify each answer to one of the three classes: [positive, neutral, negative] and add the category to each row. Add quotation marks if any answer is not in quotation marks. The answers are provided as a csv. |
| Prompt to ChatGPT o1 and Gemini 2.0 Flash Think to classify answers of Q7 – Scenario C | Based on the given answers to the question: “Do you think that within 10 years, robots like this could serve as a replacement of existing screen-based forms of entertainment such as television? Please elaborate on why or why not”, classify each answer to one of the three classes: [positive, neutral, negative] and add the category to each row. Add quotation marks if any answer is not in quotation marks. The answers are provided as a csv. |
